# Peptide array–based interactomics

**DOI:** 10.1007/s00216-021-03367-8

**Published:** 2021-05-03

**Authors:** Daniel Perez Hernandez, Gunnar Dittmar

**Affiliations:** 1 Proteomics of Cellular Signalling, Department of Infection and Immunity, Luxembourg Institute of Health, 1 A Rue Thomas Edison, 1445 Strassen, Luxembourg; 2 Department of Life Sciences and Medicine, University of Luxembourg, 4367 Belvaux, Luxembourg

**Keywords:** PRISMA, Proteomics, Interactomics, PTM, IDR, SLiM

## Abstract

The analysis of protein-protein interactions (PPIs) is essential for the understanding of cellular signaling. Besides probing PPIs with immunoprecipitation-based techniques, peptide pull-downs are an alternative tool specifically useful to study interactome changes induced by post-translational modifications. Peptides for pull-downs can be chemically synthesized and thus offer the possibility to include amino acid exchanges and post-translational modifications (PTMs) in the pull-down reaction. The combination of peptide pull-down and analysis of the binding partners with mass spectrometry offers the direct measurement of interactome changes induced by PTMs or by amino acid exchanges in the interaction site. The possibility of large-scale peptide synthesis on a membrane surface opened the possibility to systematically analyze interactome changes for mutations of many proteins at the same time. Short linear motifs (SLiMs) are amino acid patterns that can mediate protein binding. A significant number of SLiMs are located in regions of proteins, which are lacking a secondary structure, making the interaction motifs readily available for binding reactions. Peptides are particularly well suited to study protein interactions, which are based on SLiM-mediated binding. New technologies using arrayed peptides for interaction studies are able to identify SLIM-based interaction and identify the interaction motifs.

**Graphical abstract:**

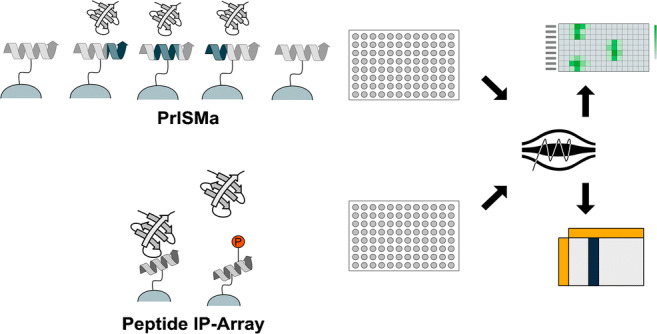

## Introduction

Cellular signaling is in large parts based on a complex network of protein interactions with other proteins or other biological molecules. Studying protein-protein interaction (PPI) networks is pivotal for the understanding of cellular signaling [1–3]. Protein-protein interactions can be studied in different ways, including the genetic modification of the protein sequence, measurements by two-hybrid interactions, or chromatographic comigration [4–6]. The direct detection of interaction partners after isolating the protein of interest is the most common one [7]. The mass spectrometric measurement of interacting proteins has increased in popularity due to the increased sensitivity and possibilities of modern mass spectrometry–based proteomics [6, 8–10]. While many studies use whole proteins for interaction studies, the use of peptides in such studies has increased over time [11–16]. In this article, we will focus on the newly developing field of peptide array–based interaction studies.

## Immunoprecipitations and peptide pull-downs

**Fig. 1:**
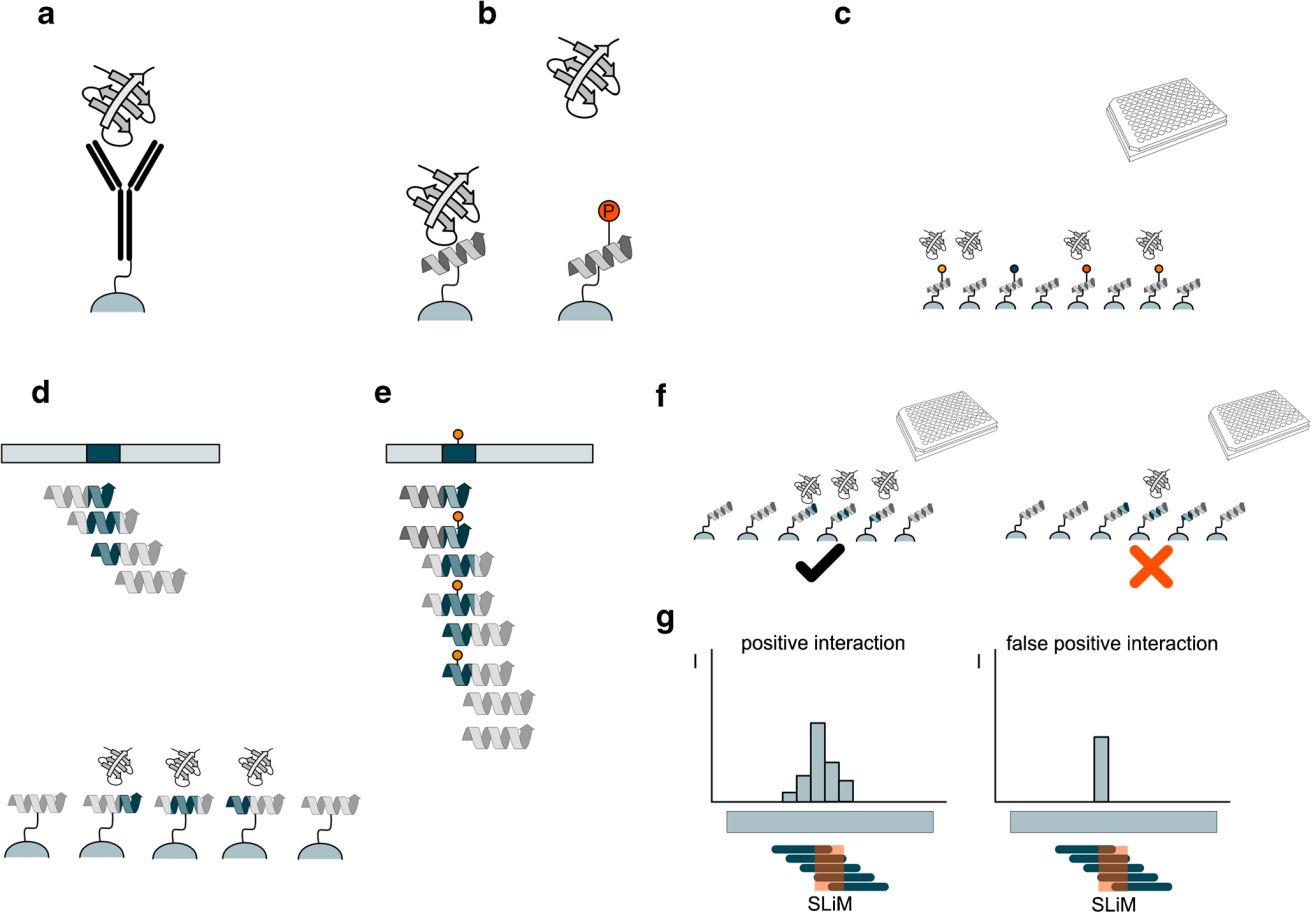
**a** Protein immunoprecipitation. The protein of interest is captured by an immobilized antibody for the pull-down. **b** Peptide pull-down. A peptide and a PTM-modified version of the same peptide are immobilized on a bead. The PTM prevents in this case the binding of the protein. **c** Highly parallelized peptide pull-down. The peptides are carrying different PTMs or mutations (indicated by the colored pins), which are enabling (orange and red) or preventing (black) interaction. **d** Peptide array designed for a PrISMa screen. A SLiM-containing area in a protein of interest (dark blue) is covered by different tiling peptides. Each of the peptides covers a different part and only the second peptide contains the entire SLiM (dark blue shade). **e** Inclusion of PTMs in the peptide array designed for PrISMa analysis. For each of the tiling peptides, a peptide with and one without the PTM is included. **f** + **g** Identification of false-positive binders in a PrISMa setup. Neighboring peptides cover different parts of the SLiM allowing only partial binding. A protein peptide not showing this binding behavior is excluded as it has a high probability to be nonspecific binding

Since its development in the early 1980s, immunoprecipitations have increasingly been used to study PPIs [17]. Using specific antibodies against the protein of interest allowed the rapid isolation and detection of interaction partners by western blotting. Despite its advantage, the technique required additional knowledge about the interaction partners, so a proper antibody could be selected (Fig. 1a). This strategy to detect PPI turned out to be of particular interest when it was combined with the introduction of protein tags as fusion proteins, using molecular biology techniques. Using fusions for all proteins in an organism allows the systematic analysis of PPI in an arrayed manner by combining protein pull-down techniques with mass spectrometric identification of the interactors [18].

In parallel, advances in the chemical synthesis of peptides allowed the production of larger quantities and longer peptides. This enabled the use of peptides in pull-down assays as an alternative to the genetic generation of tagged protein fragments [19]. The chemical synthesis of peptides carrying amino acid exchanges provided a fast alternative to cloning techniques [20–22]. Several studies used arrays of synthetic peptides containing alanine exchanges for the corresponding amino acids as a binding matrix. Incubation of the matrix and detection of the protein interactors allowed the identification of essential amino acids for the interaction [23–26]. An alternative technique for the screening for the best interacting peptides is the phage display technique, where peptides are presented on a cell using phage expressing protein fragments [21, 27–29].

## Peptide pull-down meets mass spectrometry

The full potential of a peptide pull-down is only utilized in combination with a powerful detection methodology, which allows the identification of new interaction partners. With the rise of mass spectrometry–based proteomics, interacting proteins can be identified in a peptide pull-down (reviewed in [13])*.* Differently from protein-based interaction studies, peptide-protein interactions are usually of low affinity, thus preferring high-affinity interactions with more abundant proteins [30]. This preference introduces a bias against the detection of low abundant interaction partners or transient interactions and thus limits the use of peptide pull-downs. At the same time, a peptide bait permits narrowing the interaction site down to a fraction of a protein, allowing the precise mapping of the interaction site without the need to generate protein truncations [31, 32].

A significant advantage of peptides over protein pull-downs is the possibility of including post-translational modifications (PTMs) in the peptide. As peptides are chemically synthesized, any PTM can be included as long as it is compatible with the synthesis technique. The binding surface is thus fully modified, which is usually not the case with complete proteins [33–36].

Fast regulation of biological networks relies on the rapid addition and removal of PTMs during signaling, leading in many cases to the formation or loss of protein interactions. Capturing these transient interactions is challenging [36, 37]. Dissecting the recruitment of proteins using PTM-containing peptides allows identifying different complexes involved in the interaction (Fig. 1b). The interaction of SH2 domains with the phosphorylated C-terminal tail of the epidermal growth receptor is such an example. Synthetic phosphotyrosine-containing peptides were used to show the specificity of Grp2’s SH2 domains [38, 39]. To discriminate the different interactomes of the ErbB receptors for the phosphorylated versus the unphosphorylated state, phosphorylated peptides and their unmodified counterparts derived from the C-terminal region of ErbB receptors were used in a pull-down study, revealing the specific recruitment of Stat5 to the double phosphorylated C-terminus [40]. A systematic study of all 99 human SH2 domains probed their specificity in a system biological study. The binding patterns were confirmed by peptide pull-down experiments showing the regulation in a time-resolved manner [41]. Besides phosphorylations, other PTMs have been shown to change interactions. An example is the methylation at position three of the transcription factor C/EBPß, which leads to the loss of interaction with the SWI/SNF complex [42–44].

## Linear motifs as interaction mediators

Many proteins adapt a defined fold, which is determined by their amino acid sequence. These folded domains are contrasted by regions lacking a specific fold, which are called intrinsically disordered regions (IDRs) [45]. Despite their lack of structure, IDRs are important docking sites for many proteins [46, 47]. They are often decorated with many PTMs indicating their importance for regulated interactions [45, 48–50]. IDRs can harbor many interaction motifs, which fall into three groups: short linear motifs (SLiMs), molecular recognition features (MoRFs), and intrinsically disordered domains (IDDs). The groups differ in length and how they support interactions. SLiMs are usually between three and 10 amino acids in length while MoRFs are slightly longer with 10 to 70 amino acids. MoRFs undergo a transition from the unordered to an ordered state while the ligand-binding takes place [51]. IDDs fold upon interaction with the binding partner [52].

**Fig. 2:**
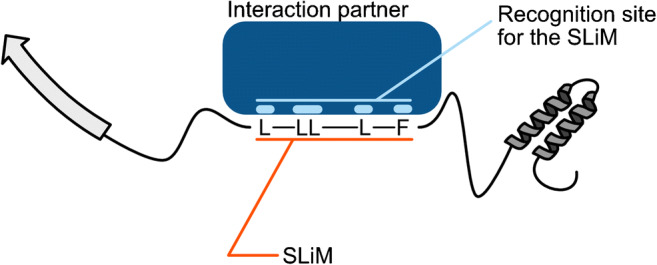
Interaction of a SLiM with a specific binding site of an interacting protein. The SLiM consists of a specific amino acid pattern, which, in this case, is defined for a set of amino acids and interspaced with amino acids (X) with no contribution to the binding (here: LXXLLXXXLXXF)

SLiMs promote interactions via specific amino acid patterns, which are recognized by their interaction partners (Fig. 2). Because of their short dimensions, SLiMs represent a compact module that is structurally and functionally autonomous [53–59]. Several SLiMs within a protein can mediate the interaction with a large variety of proteins. The compact structure and defined amino acid patterns make SLiMs preferred targets for PTM modification. A single PTM can mask a SLiM and thus prevent or mediate binding [11, 60–62].

Synthesized peptides covering short parts of a protein can contain complete SLiMs, making them the perfect carrier for SLiM-based interaction studies. This has been used in studies, where a known sequence with and without the PTM was used to identify the interaction partners, as demonstrated in a study using two different peptides derived from EGFR and Sos1 and 2. The peptides were synthesized in phosphorylated and unmodified form and used in a pull-down experiment utilizing SILAC-labeled (stable isotope labeling in cell culture) cell extracts. The interaction partners were identified using mass spectrometry [63].

## Peptide array–based interaction screens

The advancements of peptide synthesis by SPOT synthesis allow the creation of arrays holding many different peptides on a single membrane surface [23, 64, 65]. Cellulose membranes are an attractive alternative support to bead-based technologies, which can directly be used in biological assays, including immunological assays or parallelized peptide pull-downs [66]. The synthesis allows the inclusion of different PTMs in the membrane, permitting the systematic comparison of the interactome of a peptide sequence and its modified counterpart.

While peptide matrices have been used to find the optimal binding sequence [67–69], the true power of the approach emerges when it is combined with a proteomics readout. Meyer and coworkers use this principle to analyze mutations that cause neurological diseases [12]. One hundred twenty known disease-causing mutations were selected in extensive bioinformatics analysis. Peptides for the wild-type and mutated sequence were used to construct a peptide array and probed for differential binding with a proteomics readout (Fig. 1c). This created a PPI network of gained and lost interactions. A subnetwork of five interactors was related to clathrin-mediated transport. Three of the interaction nodes created a dileucine motif which is necessary for clathrin-dependent transport. In case of the glucose transporter GLUT1, the mutated version was wrongly localized to the endocytic compartment [12].

A screening technique targeting transient SLiM-based interactions along the primary structure was recently developed. The technique, PrISMa (Protein Interaction Screen on a Peptide Matrix), is based on a membrane-bound array of overlapping peptides, spanning the entire sequence of the protein of interest, creating a sliding window for the detection of SLiM-mediated interactions [11] (Fig. 1d, f). The interaction partners for each peptide were identified using mass spectrometry–based proteomics. The PrISMa technique provides a number of advantages over peptide pull-downs. The high concentration of immobilized peptides on the matrix allows the stabilization of the transient interactions, thus increasing the sensitivity for detecting these interactions. The overlapping peptide structure of the matrix offers the implementation of powerful filtering methods, based on the partial presence of the SLiM in the peptide adjacent to the main binding peptide. This allows separating unspecific background binding from SLiM-mediated interactions (Fig. 1g). The technique was used to map the interactome of the transcription factor C/EBPβ, identifying a large number of new interaction partners [11].

Additionally, the setup of the peptide array allows the inclusion of PTMs in the matrix to probe simultaneously for PTM-mediated interactions (Fig. 1e). For the C/EBPß study, several PTMs including methylation, citrullination, acetylation, and phosphorylation were included. This revealed the binding of the TLE3 complex specifically to the methylated form of C/EBPß.

A limitation of the method is the restriction of the screen: it can be only applied to intrinsically disordered regions, in which the structure depends only on the amino acid sequence. For interactions that depend on three-dimensional structures, this cannot be applied. To address this limitation, Ramberger and coworkers combined the PrISMa method with a BioID interaction screen to explore the C/EBPα interactome. They observed a significant overlap of the interaction partners between both technical approaches, and interestingly, common protein binders with C/EBPβ interactome [60].

## Outlook

Over time, peptide-based interaction studies have significantly helped to reveal interaction sites or confirm specific PTM-regulated interactions. The new matrix-based interaction screens open a new era of interaction studies allowing to test many SLiM-based interactions at the same time. Besides the costs for the peptide matrix, using a mass spectrometric readout, the time of the measurement for all the interaction screens is a major restriction. Although the measurement of certain interactions can be parallelized by the use of isotopic labeling techniques, like SILAC and maybe TMT/iTRAQ in the future, the measurement of a peptide matrix can consume weeks on data acquisition in the mass spectrometer. Here, the technical developments of fast liquid chromatography systems might reduce the time constraints and open the use of peptide matrices for more laboratories. This will broaden the use of these techniques to identify more, potential druggable, PPI-driven diseases and will promote the deeper understanding of PPI networks, which depend on low affinity interactions.
